# A Global Perspective on Drinking-Water and Sanitation Classification: An Evaluation of Census Content

**DOI:** 10.1371/journal.pone.0151645

**Published:** 2016-03-17

**Authors:** Weiyu Yu, Nicola A. Wardrop, Robert E. S. Bain, Yanzhao Lin, Ce Zhang, Jim A. Wright

**Affiliations:** 1 Geography and Environment, University of Southampton, Southampton, Hampshire, United Kingdom; 2 United Nations Children's Fund, New York City, NY, United States of America; 3 Resource Environment and Earth Science, Yunnan University, Kunming City, Yunnan Province, China; Cardiff University, UNITED KINGDOM

## Abstract

Following the recent expiry of the United Nations’ 2015 Millennium Development Goals (MDGs), new international development agenda covering 2030 water, sanitation and hygiene (WASH) targets have been proposed, which imply new demands on data sources for monitoring relevant progress. This study evaluates drinking-water and sanitation classification systems from national census questionnaire content, based upon the most recent international policy changes, to examine national population census’s ability to capture drinking-water and sanitation availability, safety, accessibility, and sustainability. In total, 247 censuses from 83 low income and lower-middle income countries were assessed using a scoring system, intended to assess harmonised water supply and sanitation classification systems for each census relative to the typology needed to monitor the proposed post-2015 indicators of WASH targets. The results signal a lack of international harmonisation and standardisation in census categorisation systems, especially concerning safety, accessibility, and sustainability of services in current census content. This suggests further refinements and harmonisation of future census content may be necessary to reflect ambitions for post-2015 monitoring.

## Introduction

Following the expiry of the United Nations’ Millennium Development Goals (MDGs) in 2015, the Open Working Group of the General Assembly has now agreed Sustainable Development Goals (SDGs) for the United Nations’ post-2015 development agenda [1]. The SDGs include a dedicated water and sanitation goal (Goal 6) with two targets on water, sanitation and hygiene (WASH) for the year 2030. The World Health Organization (WHO) and United Nations Children’s Fund (UNICEF) have been responsible for water supply and sanitation related MDG monitoring via the Joint Monitoring Programme for Water Supply and Sanitation (JMP), which organised a series of consultations working on post-2015 WASH targets and corresponding indicators [2,3]. The current proposals are built upon existing monitoring and shortcomings of the pre-2015 system and now consider water quality, reduction in inequalities between population groups, levels of service, access to basic services, settings beyond the household (schools and health centres), service sustainability, and hygiene. Four post-2015 targets with corresponding indicators and definitions were first proposed in the second WHO / UNICEF consultation [3] and subsequently refined (see Table 1) [4,5]. For assessment of the MDG targets relating to water and sanitation, the JMP monitored the proportion of population using ‘improved’ and ‘unimproved’ sanitation facilities and water supplies (definitions 1.1 and 1.2 in Table 2). This distinction is still proposed as the basis of new post-2015 definitions, but these now also incorporate accessibility, availability and quality. The new proposals additionally incorporate safety (target 3 in Table 1) and inequalities (target 4, Table 1), reflecting water and sanitation as a human right [6]. Such international policy changes will therefore place new demands on data sources for monitoring.

**Table 1:** Proposed post-2015 targets and indicators for international monitoring of access to water, sanitation and hygiene [4,5].

Target	Indicator
1. To eliminate open defecation	**1.1 Percentage of population practicing open defecation**
2. To achieve universal access to basic drinking water, sanitation and hygiene for households, schools and health facilities	**2.1 Percentage of population using ‘basic’ drinking water**; **2.2 Percentage of population using ‘basic’ sanitation**; 2.3 Percentage of population with ‘basic’ handwashing facilities with soap and water at home; 2.4 Percentage of pupils enrolled in primary and secondary schools providing basic drinking water, basic sanitation, handwashing facilities with soap and water, and menstrual hygiene management facilities; 2.5 Percentage of beneficiaries using health facilities providing basic drinking water, basic sanitation, handwashing facilities with soap and water, and menstrual hygiene management facilities.
3. To halve the proportion of the population without access at home to safely managed drinking water and sanitation services	**3.1 Percentage of population using a ‘safely managed’ drinking water service**; **3.2 Percentage of population using a ‘safely managed’ sanitation service**
4. To progressively eliminate inequalities in access	No indicator specified. This target applies to population sub-groups (rich and poor, urban and rural, slums and formal urban settlements, disadvantaged groups and the general population) for all other targets.

Indicators and targets for drinking water and sanitation where census data are particularly relevant are highlighted in bold.

**Table 2:** Definitions of improved, basic and safely managed water and sanitation facilities, as proposed for international monitoring [5,7].

	Definition	
	Drinking-water	Sanitation
1. ‘improved’	1.1 ‘improved drinking-water’: use of the following facilities: (1) piped water, (2) public tap / standpipe, (3) tubewell / borehole, (4) protected dug well, (5) protected spring, (6) rainwater	1.2 ‘improved sanitation’: exclusive use by a single household of the following facilities: (1) ventilated improved pit latrine, (2) pit latrine with slab, (3) composting toilet, and flush / pour facility draining to (4) sewer, (5) septic tank, or (6) pit latrine, and (7) special cases (e.g. flush / pour to unknown place or not sure or DK where)
2. ‘basic’	2.1 ‘basic drinking-water’: use of ‘improved drinking-water’ with a total collection time of no more than 30 minutes for a roundtrip including queuing	2.2 ‘basic sanitation’: could be any of the following limited sharing categories (shared among no more than 5 families or 30 persons, whichever is fewer, and if users know each other): (1) ventilated improved pit latrine, (2) any pit latrine with a superstructure, and a platform or squatting slab constructed of durable material, (3) composting toilet, and flush / pour facility draining to (4) sewer (small bore or conventional), (5) septic tank, or (6) pit latrine.
3. ‘safely managed’	3.1 ‘safely managed drinking-water’: use of a water source at the household or plot which reliably delivers enough water to meet domestic needs, complies with WHO Guideline Values for *Escherichia coli* (*E*. *coli*), arsenic and fluoride, and is subject to a verified risk management plan, could be the following facilities: (1) piped water, (2) public tap / standpipe, (3) tubewell / borehole, (4) protected dug well, (5) protected spring, (6) rainwater; and could potentially be (7) delivered water (e.g. by truck, cart, sachet, or bottle)	3.2 ‘safely managed sanitation’: use of a ‘basic sanitation’ facility by which the excreta is safely transported to a designated disposal / treatment site, or treated in situ before being re-used or returned to the environment

Alongside household surveys, population censuses are one of the data sources currently used for international monitoring and 252 censuses were included in the JMP database by 2014 [8]. Being based on near complete population enumeration, they provide some advantages over nationally representative surveys, such as Demographic and Health Surveys. With their full population coverage, census data can be spatially disaggregated to a greater extent than survey data [9] and enable water and sanitation access to be quantified even for small minority populations. A global trend towards greater access to improved water sources and sanitation [4] means the proportions of those without safe water and adequate sanitation are becoming smaller in most countries. Simultaneously, there is an emerging policy emphasis on monitoring inequalities in access among minority population groups [3], reflected in target 4 in Table 1. Together, these developments mean it is likely to become increasingly expensive to statistically power household surveys to monitor inequalities in safe water access, as larger sample sizes will be required.

However, whilst Demographic and Health Surveys include core, standardised questions on water and sanitation [10], census questions on water and sanitation are generally less standardised. The United Nations Department of Economic and Social Affairs issues recommendations on implementation of censuses [11], including assessment of housing, but does not require inclusion of core questions on water and sanitation. Inconsistent census terminology, for example due to different national circumstances and priorities, may undermine its utility for international monitoring, such as monitoring progress in universal basic drinking-water and adequate sanitation. There are attempts to address this issue. The Integrated Public Use Microdata Series International (IPUMS-I), developed by the Minnesota Population Center, University of Minnesota, harmonises national census data spatio-temporally to enable a universal classification system of variables across countries and time [12]. IPUMS-I harmonisation differentiates piped water versus other supply types and flush toilets versus other sanitation. Moreover, their experience suggests the harmonisation of terminologies in census data can be challenging [13], due to uncertain meanings caused by cultural differences, uneven data quality, and the large number of samples and variables which requiring standardisation. The JMP have also harmonised water and sanitation-related terminology as part of international monitoring efforts. In many instances, the JMP apply adjustment corrections [8] to water supply types that encompass both ‘improved’ and ‘unimproved’ supply types, for example to estimate the proportions of protected and unprotected wells within an undifferentiated category of ‘wells’. International variation in census questionnaire content has been studied for other population characteristics: for example, Morning [14] analysed the 2000 census round questionnaire content on ethnic classification systematically and found that the terminology for ethnicity varied by world region. However, to date there has been no study of the water and sanitation-related content of censuses.

This study, therefore, aims to assess international and temporal variation in water and sanitation-related census content within selected low and lower-middle income countries, within the context of post-2015 changes to international monitoring. Higher income countries are excluded, since they typically have more limited census content on water and sanitation.

## Methodology

### Study countries

Study countries comprised the current 34 low income and 50 lower-middle income countries [15]. Of these, 34 countries were assessed as not having met 2015 targets for drinking-water, and 61 countries as not having met 2015 targets for sanitation (where data are available), according to the most recent JMP report [16]. South Sudan was excluded because no census had been conducted since independence in 2011.

### Data sources

Where possible, copies of census questionnaires were acquired from IPUMS-I, which contained more than 1,000 national census questionnaire forms, and also provided supplementary documents (e.g. enumerator instruction manuals) via its subordinate portals, such as the African Integrated Census Microdata (AICMD) portal. For a minority of countries lacking census materials from IPUMS-I, questionnaire and related content was obtained from other international sources, such as the United Nations Statistics Division (UNSD), National Statistical Offices (NSOs), or other organisations, wherever available.

### Census questionnaires and materials

For questionnaires in languages other than English, census content was characterised using questionnaires translated into English and provided by the IPUMS-I, UNSD, or other sources such as NSOs wherever possible. For those census questionnaires which were not available in English, content was translated by native speakers. In addition, long format questionnaires (when available) were used rather than short format questionnaires since they are likely to be more detailed in content. Census questionnaires were supplemented by implementation manuals, enumerator instructions, and / or IPUMS-I explanatory notes for harmonisation, alongside information from JMP country files, which contain tables of source data with detailed classifications for drinking-water source and sanitation facility. In addition, JMP country files were used to guide the harmonisation of census questionnaire content relevant to drinking-water and sanitation.

### Content analysis framework

#### Harmonisation of census content for monitoring progress towards post-2015 targets

A scoring system was developed to assess potential issues in harmonising census questionnaire responses for monitoring progress towards post-2015 WASH targets, as detailed in Tables 3 and 4. Questionnaires which contained no content on household water and sanitation scored zero. For all other questionnaires, harmonised water supply and sanitation services for each census were assessed relative to the typology needed to monitor the proposed post-2015 indicators in Table 1. For water access, four component of census content were scored (Table 3): content on improved water sources (W_1_); content relating to water collection times (W_2_); whether improved water sources were on a household’s premises (W_3_); and whether the census covered supply interruptions (W_4_). Each component was scored as a proportion from zero to one. For W_2_, W_3_ and W_4_, which involved the proportion of improved source type categories, we included potentially ambiguous improved sources (typically wells and springs) in the denominator and also in the numerator for W_3_ when calculating proportions. Any type of ‘delivered / vended water’ such as via bottle, barrel or tank was considered as unimproved drinking-water if unspecified. ‘Other(s)’, ‘not stated’ and ‘don’t know’ were not included when calculating proportions. To reduce ambiguity in interpretation of questionnaire content, a detailed protocol was developed to support consistent interpretation of terminology and question wording (S1 Text). Some post-MDG indicator elements, such as water quality or water safety plans, were not scored, as they are absent from all censuses and require other data streams for monitoring.

**Table 3:** Scoring system for assessing suitability of census questionnaire content for monitoring progress towards post-2015 targets relating to water.

Indicator (Table 1) definition of post-2015 targets	Corresponding scoring of census questionnaire content
Indicator 2.1: Percentage of population using water from an **improved source** with a **total collection time of 30 minutes or less for a roundtrip, including queuing**	(1) Score W_1_: the proportion of water source categories that can be unambiguously distinguished as either improved or unimproved; (2) Score W_2_: the proportion of off-premises improved water source categories for which collection time or related information (e.g. distance to water source) is available.
Indicator 3.1: Percentage of population using a water source **at the household or plot** which **reliably** delivers enough water to meet domestic needs, complies with WHO Guideline Values for *E*.*coli*, arsenic and fluoride, and is subject to a verified risk management plan	(1) Score W_1_: the proportion of water source categories that can be unambiguously distinguished as either improved or unimproved; (2) Score W_3_: the proportion of improved water source categories that can be unambiguously distinguished as either on premises or off premises; (3) Score W_4_: the proportion of improved water source categories for which information about water supply interruptions (e.g. in days) is available.

Indicator components relevant to each criterion score are highlighted in bold.

**Table 4:** Scoring system for assessing suitability of census questionnaire content for monitoring progress towards post-2015 targets relating to sanitation.

Indicator definition of post-2015 targets	Corresponding scoring of census questionnaire content
Indicator 1.1: Percentage of population **practicing open defecation**	Score S_1_: those with no sanitation facility can be unambiguously distinguished from those with facilities (1 = true, otherwise 0).
Indicator 2.2: Percentage of population **using a sanitation facility that effectively separate excreta from human contact, and ensure that excreta do not re-enter the immediate household environment**, **and the facility is shared among no more than 5 families or 30 persons, whichever is fewer, and if the users know each other**	(1) Score S_2_: ‘basic’ sanitation categories can be unambiguously distinguished from ‘non-basic’ sanitation types (1 if true; 0.5 if true for some categories; otherwise 0); (2) Score S_3_: ‘basic’ sanitation facilities can be unambiguously distinguished as private, shared (not public), and public (1 if true for three categories–‘private’, ‘shared’ and ‘public’; 0.5 if true for two categories; otherwise 0).
Indicator 3.2: Percentage of people who (1) use a **‘basic’ sanitation facility** and (2) whose **excreta is safely transported to a designated disposal / treatment site, or treated in situ before being re-used or returned to the environment**	(1) Score S_2_: ‘basic’ sanitation categories can be unambiguously distinguished from ‘non-basic’ sanitation types (1 if true; 0.5 if true for some categories; otherwise 0); (2) Score S_3_: ‘basic’ sanitation facilities can be unambiguously distinguished as private, shared (not public), and public (1 if true for three categories–‘private’, ‘shared’ and ‘public’; 0.5 if true for two categories; otherwise 0); (3) Score S_4_: sanitation facilities have information about the elimination or disposal of excreta (1 if true; 0.5 if true for some categories; otherwise 0).

Indicator components relevant to each criterion score are highlighted in bold.

For sanitation, again four components of census content were scored (Table 4): open defecation (S_1_); ‘basic’ sanitation categories (S_2_); use of shared sanitation (S_3_); and excreta removal from household or disposal (S_4_). We assumed for S_3_ when both ‘shared’ and ‘public’ sanitation were used as response options, ‘shared’ referred to ‘limited sharing’ (i.e. a sanitation facility shared by more than one family but less than five families or 30 persons); whilst ‘public’ sanitation referred to a sanitation facility shared by more than 5 families or 30 persons. Initial piloting of the content analysis framework suggested sanitation-related census content was typically more complex and entailed multiple questions, leading to inconsistent interpretation of proportions. Therefore, a simpler scoring system was used whereby each criterion scored one if fully met, 0.5 if partially met, and zero if not met. In interpreting content concerning wastewater disposal, we assumed the same arrangements applied to both grey water and sewage, but ignored solid waste disposal arrangements. For both drinking-water and sanitation scoring system, ‘other(s)’ was considered as an ‘undistinguishable’ sanitation type.

Where there was uncertainty or subjective judgements were required, these issues were recorded and discussion between five of the co-authors (WY, NAW, JAW, CZ, and YL) used to reach an agreement.

#### Analysis of water and sanitation scores

The resultant water and sanitation (W/S) scores from the census content scoring system were analysed spatio-temporally. Given that other census content areas, for example ethnicity, were found to vary by world region [14], the W/S-scores were aggregated by six world regions [17] to explore inter-regional variation in the water and sanitation items in census content: East Asia and Pacific (EAP); Europe and Central Asia (ECA); Latin America and Caribbean (LAC); Middle East and North Africa (MENA); South Asia (SOA); and Sub-Saharan Africa (SSA). The W/S-scores were assessed by census round to monitor progress in census content development concerning drinking-water and sanitation over time. A census round refers to those national population and household censuses carried out within ten year intervals; for instance, the 2000 round census refers to censuses carried out from 1995 to 2004. For those countries with more than one census in a given round, we selected the most recent one. Since fewer countries were characterised in earlier census rounds, we used t-test paired by country to test for significant increases in W/S-scores between successive census rounds, alongside Cohen’s d to determine the effect size. T-test and Cohen’s d were also used to identify significant differences in scores between regions. Box plots by region were employed to examine the distribution of W/S-scores.

#### Characterisation of other census questionnaire content

Additional questionnaire response categories and other questionnaire characteristics (Table 5) of relevance to international monitoring were recorded but not scored. Characterisation assessed whether the following common categories could be distinguished: piped water, tubewell / borehole, well, spring, rainwater, tanker-truck / cart with small tank or drum, bottled water, and surface water for drinking-water; and flush / pour-flush, ventilated improved pit (VIP) latrine, pit latrine, composting toilet, bucket, hanging toilet, and open defecation for sanitation. Where these categories were combined in a single response option (e.g. protected well and spring, springs and surface water, vended water, etc.), they were not considered distinguishable.

**Table 5:** Other unscored population census characteristics of relevance to WASH targets.

Characteristics	Detailed information
Questionnaire section	Household section of questionnaire versus individual section
Actual usage of facility	‘Access to facility’ versus ‘use of facility’
Multiple sources / facilities	(1) ‘Source / facility’ versus ‘main source / facility’ (and secondary source / facility, if available); (2) ‘Dry season’ versus ‘wet season’
Water use purpose	‘For drinking (and / or cooking) purposes’ versus ‘for other domestic purposes’
Hygiene information	e.g. soap, hand-washing, bath / shower, etc.

### Ethics Statement

This study only analysed the contents of openly accessible historical census questionnaire forms; the authors did not actively collect any new data, and the study did not entail any work with human subjects nor collect any data via questionnaires; it therefore did not involve any ethical concerns.

## Results

**Fig 1:**
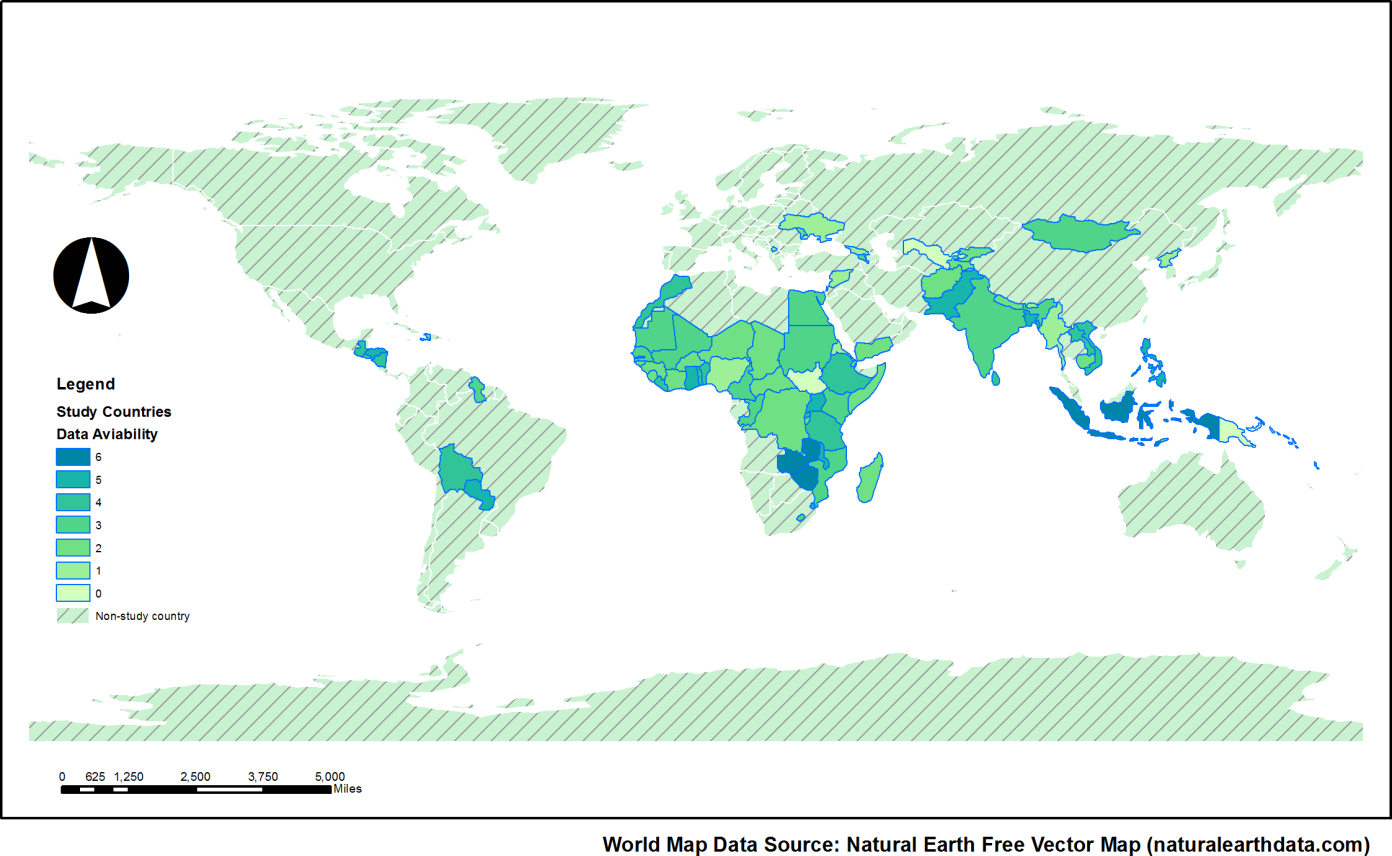
Census questionnaire availability for 83 low and middle income countries. Colours indicate the number of census rounds with accessible questionnaire materials. The world map is sourced from Natural Earth vector map data.

Overall, 247 questionnaires (68.6% of the 360 censuses conducted in total in the 83 countries) were analysed (see Fig 1). Exclusions were mainly due to census questionnaires being unavailable (56 censuses), ambiguous or incomplete census documentation (49 censuses), or language restrictions (8 censuses) in some cases. Questionnaires shared by different countries for example due to sub-division of former national entities are counted only once in the analysis.

### Analysis of Water and Sanitation Scores

Overall, the W-scores ranged from 0 to 3 with an average value of 1.21 (median = 1.25); whilst S-scores ranged from 0 to 3 with an average value of 1.26 (median = 1.5). Detailed statistics for resultant scores are shown in ‘S2 Text’. The water collection time (W_2_) and water supply interruption (W_4_) scores are low for the 247 included censuses; most (90.3% and 99.6% respectively) scored zero for these components. In comparison, only 14.2% and 24.3% of censuses scored zero for having distinguishable improved / unimproved water source classes (W_1_) and distinguishable on / off-premises improved water source classes (W_3_) respectively. For sanitation, most (72.9%) of the questions identify open defecation. However, with regard to ‘basic’ sanitation categories (S_2_), use of shared sanitation (S_3_), and excreta elimination or disposal (S_4_), most census questionnaires generally lack sufficient information: 83.8%, 57.9% and 61.9% of the total observed questionnaires scored zero for S_2_, S_3_, and S_4_ respectively.

### Changes in census content over time

In general, the paired (by country) t-test suggested that there were significant increases over time for both water (excluding that between rounds 1 and 2; 4 and 5) and sanitation (excluding that between rounds 2 and 3) (Tables 6 and 7). Detailed scores were also separately tested for W_1_, W_3_, S_1_, and S_3_ (for which >50% of censuses scored over zero): most were not significantly changed in earlier rounds (1 and 2); W_1_ showed significant changes over all rounds after round 2; S_3_ was found to have no significant changes over time.

**Table 6:** Table of p-value for paired (by country) t-test for significant changes in water and sanitation scores between census rounds.

	W	S	W_1_	W_3_	S_1_	S_3_
R1 vs R2	0.057	**0.027**	0.072	0.055	0.083	0.096
R2 vs R3	**0.004**	0.090	**0.003**	0.173	0.134	0.358
R3 vs R4	**0.011**	**0.009**	**0.006**	**0.017**	**0.048**	0.067
R4 vs R5	0.099	**0.029**	**0.013**	0.411	0.092	0.331
R5 vs R6	**<0.001**	**<0.001**	**<0.001**	**<0.001**	**0.017**	0.162
R1 vs R4	**0.001**	**0.001**	**<0.001**	**0.001**	**0.004**	0.052
R4 vs R6	**<0.001**	**<0.001**	**<0.001**	**0.035**	0.052	0.132
R1 vs R6	**<0.001**	**0.001**	**<0.001**	**0.004**	**0.006**	0.052

P-values < 0.05 highlighted in bold; R refers to census round (e.g. R1 vs R2: paired t-test between census round 1 and round 2).

**Table 7:** Table of Cohen’s d corresponding to Table 6.

	W	S	W_1_	W_3_	S_1_	S_3_
R1 vs R2	0.419	0.429	0.447	0.412	0.344	0.426
R2 vs R3	0.864	0.373	0.875	0.254	0.319	0.097
R3 vs R4	0.343	0.622	0.527	0.286	0.435	0.371
R4 vs R5	0.240	0.297	0.434	0.041	0.231	0.062
R5 vs R6	0.634	0.768	0.680	0.590	0.434	0.123
R1 vs R4	1.705	1.058	1.656	1.368	1.035	0.641
R4 vs R6	0.817	0.781	1.021	0.456	0.393	0.186
R1 vs R6	1.808	1.314	2.989	1.220	1.319	0.549

R refers to census round (e.g. R1 vs R2: paired t-test between census round 1 and round 2).

#### Regional variability in census content

**Fig 2:**
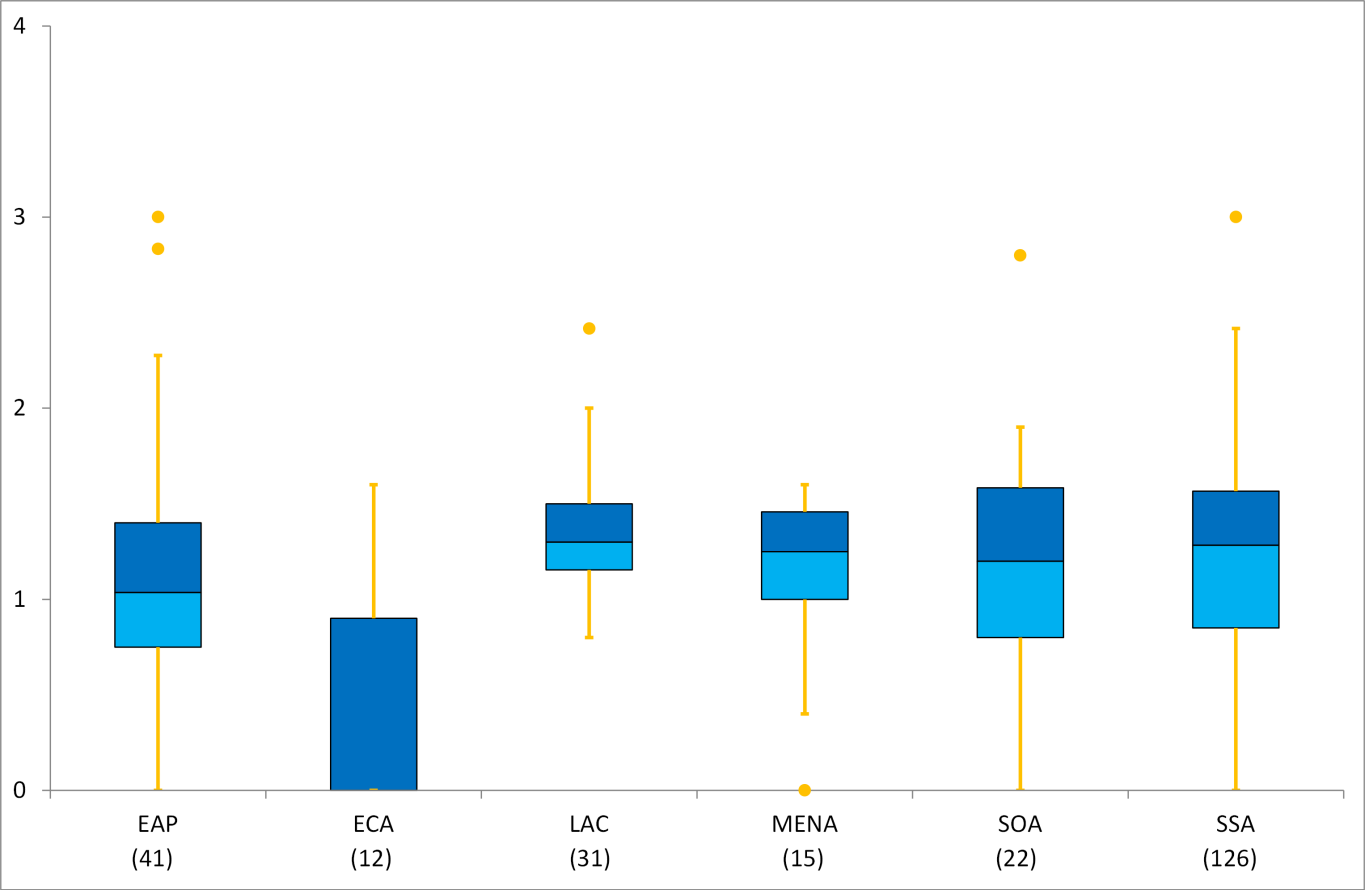
Box plots of W-scores by region. The bottom (light blue) and top (dark blue) of the box represent the 25^th^ and 75^th^ percentiles respectively.

**Fig 3:**
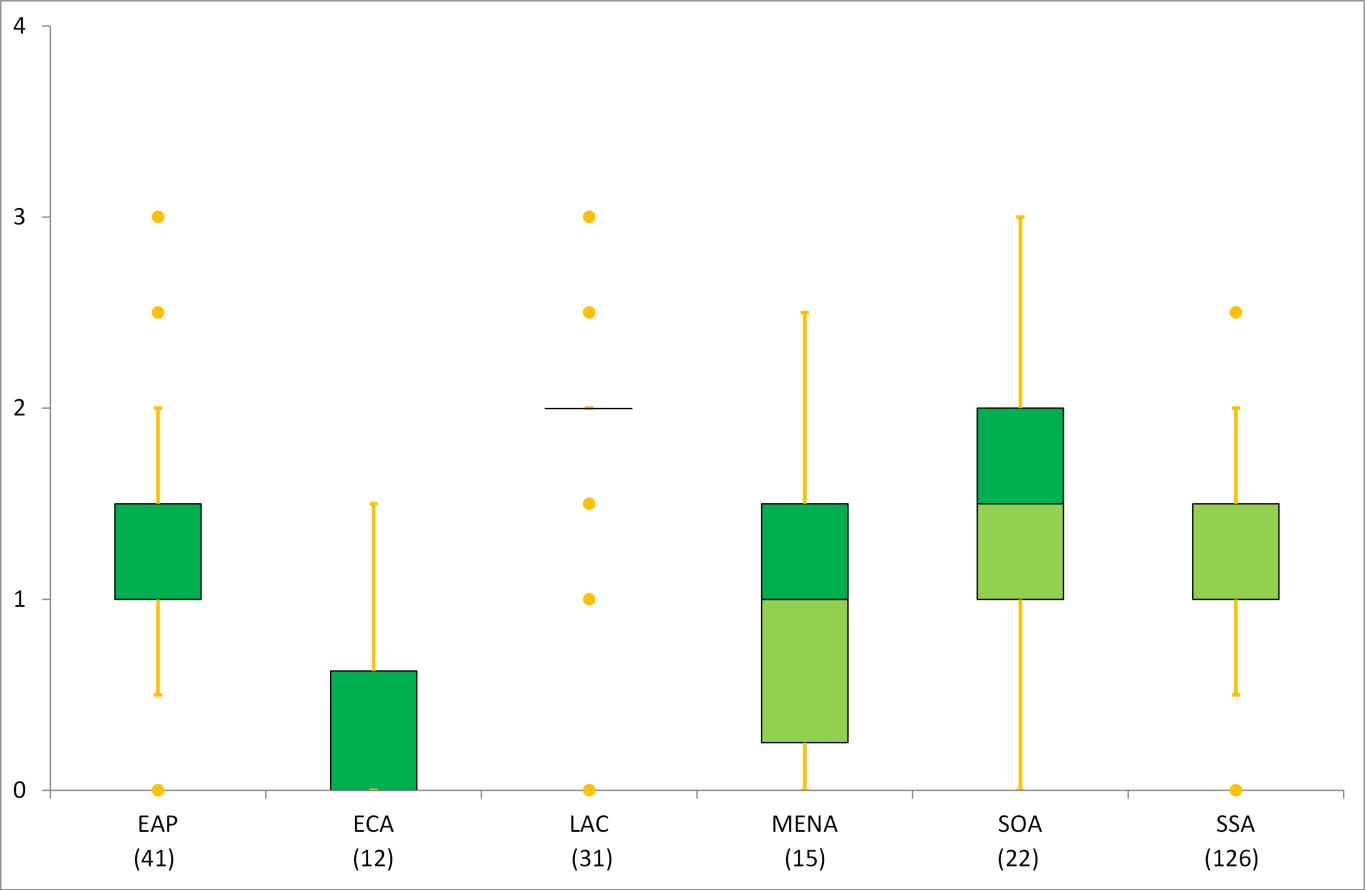
Box plots of S-scores by region. The bottom (light green) and top (dark green) of the box represent the 25^th^ and 75^th^ percentiles respectively.

In terms of spatial patterns, the regional results generally did not display any significant differences, only except the small sampled ECA (in total 12 censuses of 7 countries; most of them were independent from the Soviet Union) which had lower scores relative to other regions (Figs 2 and 3). The regions of ECA and LAC interpreted large mean effect sizes (Table 8) in comparison with other regions for the mean scores of both water and sanitation.

**Table 8:** p-values of t-tests and Cohen’s d between regions in water and sanitation scores.

Regional comparison	Water		Sanitation	
	p-value	Cohen’s d	p-value	Cohen’s d
EAP versus ECA	**0.002**	0.22	**0.003**	0.23
EAP versus LAC	0.133	0.33	**<0.001**	**0.94**
EAP versus MENA	0.456	0.23	0.481	0.21
EAP versus SOA	0.831	0.06	0.255	0.30
EAP versus SSA	0.444	0.14	0.881	0.03
ECA versus LAC	**<0.001**	**2.16**	**<0.001**	**2.59**
ECA versus MENA	**0.012**	**1.05**	**0.041**	**0.83**
ECA versus SOA	**0.002**	**1.21**	**0.003**	**1.17**
ECA versus SSA	**<0.001**	**1.11**	**0.001**	**1.03**
LAC versus MENA	**0.012**	**0.82**	**<0.001**	**1.28**
LAC versus SOA	0.335	0.30	0.112	**0.50**
LAC versus SSA	0.359	0.12	**<0.001**	**0.94**
MENA versus SOA	0.368	0.31	0.174	0.46
MENA versus SSA	0.222	0.33	0.467	0.20
SOA versus SSA	0.709	0.09	0.217	0.36

Significant differences (p-values < 0.05) and large effect size (Cohen’s d values > 0.5) highlighted in bold.

### Other content

Just over a quarter (25.9%) of the 247 census questionnaires included contained sewerage-related questions; 32.0% contained questions about hygiene (mostly about bath or shower facilities); and 24.3% contained questions about solid waste. In terms of the general questionnaire format, 19.0% asked more than one question about water and 17.8% about sanitation; 10.5% and 25.1% of censuses contained questions about use of facilities, as opposed to access, for (drinking) water and sanitation respectively. Questions concerning ‘use’ were worded differently depending on the census and country. For example, sometimes questions concerned the nature of the facility present within the home alongside the use of facility (e.g. The Gambia 1983), but sometimes respondents were asked about the main facility used by household members, regardless of its location (e.g. Liberia 2008). Questions asked about the ‘main’ facility more for water (37.7%) than for sanitation (10.9%). ‘Other(s)’, ‘not stated’ and ‘don’t know’ are widely used for water-related census content (68.4%), in comparison with sanitation (38.1%). Only 37.3% of the census questionnaires specified if the water was for drinking or other domestic uses. In addition, 11.3% of sanitation questions involved the location of sanitation (e.g. inside the house or outside). Malawi (census 1987, 1998 and 2008) is the only country that mentioned seasonal change when asking about drinking-water. There were very few (2.8%) of the observed questionnaires included different sections or forms for different population groups, including urban population versus rural population, and sedentary population versus nomadic population. Water and sanitation questions appeared in the household section of census questionnaires rather than the section about individuals in all cases.

**Fig 4:**
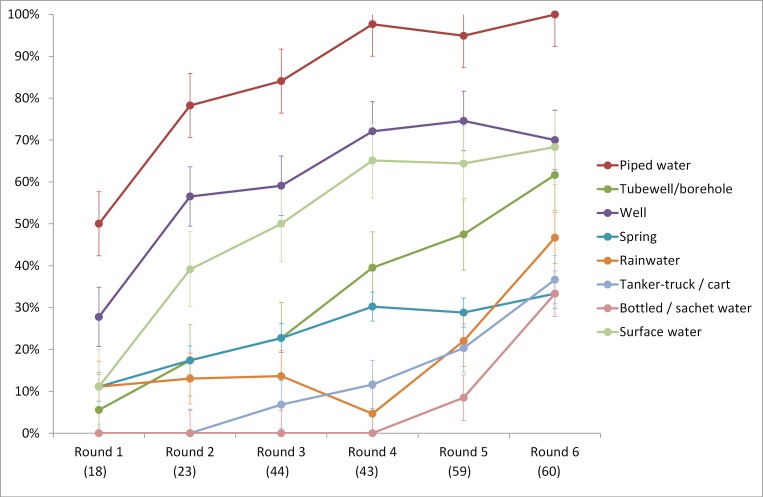
Proportion of questionnaires using common drinking-water categories in included census questionnaires by round. Y-axis represents the percentage (%) of included censuses; x-axis is the census round with corresponding number of included censuses in brackets; Round 1: 1956–1965; Round 2: 1966–1975; Round 3: 1976–1985; Round 4: 1986–1995; Round 5: 1996–2005; Round 6: 2006–2015; error bars show standard errors.

**Fig 5:**
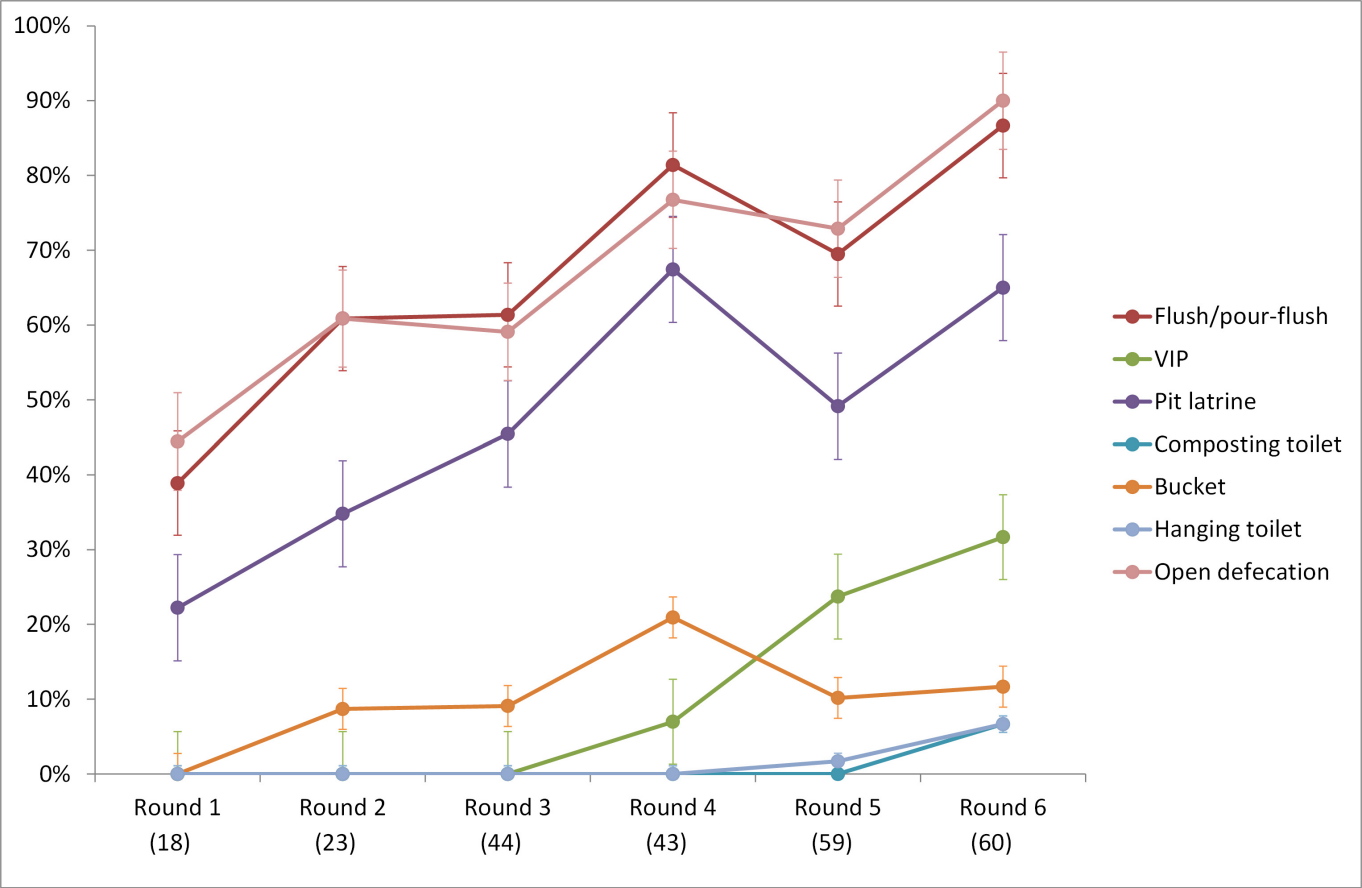
Proportion of questionnaires using common sanitation categories in included census questionnaires by round. Y-axis represents the percentage (%) of included censuses; x-axis is the census round with corresponding number of included censuses in brackets; Round 1: 1956–1965; Round 2: 1966–1975; Round 3: 1976–1985; Round 4: 1986–1995; Round 5: 1996–2005; Round 6: 2006–2015; error bars show standard errors.

Figs 4 and 5 show the proportion of censuses in each round for which the JMP-defined categories for drinking-water and sanitation can be clearly identified. Although it is difficult to detect patterns in earlier census rounds due to small sample sizes, the results suggest that the JMP defined categories can be classified as two groups in terms of their use in census content: high-use categories and low-use categories. High-use categories include piped water, well, and surface water for drinking-water, which have been used in >50% of censuses since round 3; and flush / pour-flush, open defecation, and pit latrine for sanitation, used in >50% of censuses since round 4. The other categories of both drinking-water and sanitation are classified as low-use categories, given their low percentage of usage in census (less than 50% over six rounds). ‘Tubewell / borehole’ lies between these two groups, and may have been classified under ‘well’ in earlier censuses, but has been increasingly distinguished as a category in its own right over time (over 50% in round 6).

## Discussion

### Findings

This study evaluated drinking-water and sanitation classification systems within census content based upon a scoring system developed to assess suitability for monitoring progress towards post-2015 WASH targets. In general, temporally, the resultant water and sanitation scores increased over the six census rounds; however, spatially, there was no evidence of significant differences between the six world regions, although previous evidence suggests that other content areas, such as terminology concerning ethnicity, do vary by world region [14]. For water-related content, censuses generally distinguish improved from unimproved drinking-water (W_1_) and improved sources that are on or off premises (W_3_), but few capture water collection time (W_2_) or water supply interruptions (W_4_). For sanitation, census questionnaires usually distinguish households practicing open defecation (S_1_) and using ‘basic’ sanitation (S_2_), but fewer census questions assess sharing of facilities (S_3_) and elimination or disposal of excreta (S_4_). Given that populations lacking improved drinking-water sources are increasingly concentrated in an ever smaller number of settings [4], reaching the unserved will require focussed effort and greater monitoring of minority groups. The use of conventional household surveys for this purpose tends to be expensive, because of the need for oversampling. Although household survey designs can be modified without incurring excessive costs [18], for example by oversampling minority populations, relative to conventional household survey designs that currently predominate, censuses are better able to distinguish differences in service provision among minority groups, given that they seek to fully enumerate populations. Censuses will become even more dominant in the estimates, should the JMP adopt proposals to weight data sources by a source quality metric and sample size [19]. The JMP will continue to report on water and sanitation ladders with censuses and household surveys forming the cornerstone for reporting on higher levels of service such as ‘safely managed’. In addition, census data can be used to triangulate information from regulators and utilities and to determine which population groups are covered by these new data sources for the JMP.

There has been discussion of the role of international monitoring arrangements as a normative influence informing national monitoring practice [20]. The post-2015 indicators may result in more widespread use of census questions concerning water collection times, shared sanitation, and excreta elimination or disposal. Although historically, censuses have increasingly been able to differentiate the categories used by the JMP (Figs 4 and 5), the extent to which this can be attributed to international monitoring arrangements post-2015 is unclear. There is no obvious step-change in the differentiation of these categories and water and sanitation scores for individual countries fluctuate over time, likely reflecting changing national priorities and pressure to reduce census questionnaire length. Similarly, the rapid growth in the use of some water and sanitation categories, notably packaged or bottled water, may reflect their growing importance as water sources [21].

Aside from the water and sanitation categories used, several other aspects of the WASH-related content of censuses are noticeable in these findings. Firstly, although there has been a long history of inclusion of water and sanitation questions in censuses, questions about hygiene and solid waste disposal are seldom included, though post-2015 hygiene-related targets have been proposed [5]. Secondly, despite intra-household inequalities being recognised as important [22], water and sanitation questions universally appear in the household sections of censuses. Thirdly, there is growing recognition that households often use different water sources depending on season and for different purposes [23], yet few censuses capture seasonal variation in water and sanitation use or the use of different water sources for different purposes. Whilst capturing such components of water and sanitation access in censuses would appear to be desirable, longer census questionnaires are more expensive to implement and monitoring costs need to be commensurate with budgets for programmatic delivery. There are however now several reports [24] that call for greater investment in data and monitoring in lower income countries.

### Limitations of this study

There are a series of limitations, assumptions and uncertainties affecting this study, which relate to the scoring system, the census materials used, underlying assumptions, and the broader international policy environment. Firstly, the scoring system measures the ‘distinguishability’ of sanitation or drinking-water types via a proportion of the response categories used for a census question, so as to avoid complications from specific categories which are not relevant in some countries. As a consequence, when a larger number of more detailed response categories are developed for a new round, the score can sometimes decrease because the denominator is larger, despite the richer question content. Secondly, since the W-scores are calculated as proportions but S-scores on a coarser two or three-point scale, it may not be appropriate to aggregate the two into a single composite score. Thirdly, the eight score components are weighted equally; however, their significance might be quite different. Fourthly, the scoring system used is only an approximation of international monitoring requirements embodied in post-2015 proposals, so for example, distance to water source may not reflect water collection time including queuing time.

There are three main types of material that can be used for assessing the drinking-water and sanitation classification systems in censuses: census data (either micro-data or aggregated by area), census reports (readily accessible, but presenting aggregated findings), and census questionnaires and manuals. This study examined questionnaire and manual content, but this may not reflect the water and sanitation categories used in summary reports or data, since aggregation across categories may take place prior to release of both. Similarly, where multiple water and sanitation questions are used in censuses, cross-tabulations of these questions (e.g. water source type versus distance to source) may not be directly accessible in geographically aggregated data and reports, due to for example identifiable individual protection.

The resultant scores are subject to a series of assumptions (documented in S1 Text), which are necessarily made for the interpretation of specific terminology across observers (both in interpreting words such as ‘public’ and in interpreting water and sanitation categories such as ‘tank’). In addition, the subjective interpretation of the water and sanitation items in census content is generally also dependent on question wording (e.g. ‘no toilet’ could refer to ‘open defecation’ or ‘no toilet available in dwelling’ given different context), language or translation, and the availability of supporting information in contextual materials such as manuals or additional documentation in IPUMS etc., may vary by country and individual census case.

Finally, given that proposals for post-2015 targets, indicators and corresponding definitions for international monitoring of WASH are yet to be adopted, the underlying framework for scoring used here may not reflect eventual post-2015 monitoring arrangements.

### Future research

As new post-2015 arrangements become operational, the framework developed here could potentially be used to examine the way in which international monitoring arrangements influence national monitoring practice and vice versa. Similarly, there would be scope to expand the range of census content characterised under the framework developed here, for example by documenting potential stratifiers in census questionnaires that might be suitable for examining inequalities in water and sanitation access, such as ethnicity or disabilities. Finally, given the variation in water and sanitation categories evident in this analysis, the impact of definitional ambiguities and harmonisation assumptions on census-based international comparisons of water and sanitation access could also be explored. There might also be worthy to undertake a similar exercise that analyses the terminology used in household surveys, since these too are known to vary [25] though to a lesser extent than censuses. In this regard, there would also be scope to assess uncertainty in the scores presented here via independent characterisation of content by different individuals and subsequent assessment of inter-observer agreement.

## Conclusions

This study applied a scoring system to assess the ability of census questionnaires to capture drinking-water and sanitation availability, safety, accessibility, and sustainability. Census questionnaires generally distinguish between those households with improved versus unimproved drinking-water supply types and most censuses are able to identify households practicing open defecation. This pattern of data availability is encouraging for assessment of inequalities for those lacking services altogether. However, there are important proposed post-MDG indicator elements, such as water quality, that are not present in census questions and which, consequently, could not be measured in this study. Whilst there was limited regional variation in content, there is evidence that the information content of census-based water and sanitation questions has increased since earlier census rounds, though how far this trend has been influenced by international monitoring requirements is unclear. In other respects, these findings also suggest that there are many WASH elements that census data seldom capture, such as intra-household and seasonal variations in service access, hygiene, and sharing of sanitation. Post-2015 international monitoring targets may provide a new impetus to assess such components. Despite the infrequent administration of censuses relative to household surveys (with censuses generally taking place every decade), it is expected that all 83 study countries would conduct at least one census during the 2020 census round (2016–2025). As the definitions underpinning monitoring for the SDGs have recently been finalised [26], by this stage there should be potential for harmonisation of water and sanitation terminology in national census-taking worldwide through the normative role of international monitoring arrangements. As evidenced by the limited or non-existent water quality and supply interruption components within census data, some components of proposed indicators for international monitoring such as water safety will be difficult to capture via a single data source. Going forwards, publication of geographically disaggregated water and sanitation census data is likely to become increasingly important for international monitoring if census data are to be integrated with other data streams.

## Supporting Information

S1 Text Detailed protocol for interpretation of questionnaire content. (DOCX)

S2 Text Detailed statistics of W/S-scores by census round. (XLSX)
