# Supplementary material for: A Global Perspective on Drinking-Water and Sanitation Classification: An Evaluation of Census Content
Source: PLoS One. 2016 Mar 17;11(3):e0151645. doi: 10.1371/journal.pone.0151645 (PMC4795766; doi:10.1371/journal.pone.0151645)
Supplement: S1 Text — (DOCX) [file pone.0151645.s001.docx]

# S1 Text: Detailed protocol for interpretation of questionnaire content

## Drinking-water:

(1) Neighbour (piped water)

‘Piped to neighbour(s)’: improved drinking-water, off premises;

‘Shared with neighbour(s)’: improved drinking-water, on premises or off premises.

(2) Drilled well: improved drinking-water, on premises or off premises.

(3) Hand pump: improved drinking-water, on premises or off premises.

(4) Artesian well: improved or unimproved drinking-water, on premises or off premises.

(5) Tank: improved or unimproved drinking-water, on premises or off premises.

(Unspecified ‘tank’ could be rainwater harvesting system; water tank which pumped water from underground into tank above ground and then deliver water through pipe; water tank filled by water from a tanker-truck via a pipe, etc.)

(6) ‘Private / individual / household’ equipment

Network system (original source of piped water): on premises or off premises;

Well (or any other ground water): on premises;

Tank (no matter water tank or rainwater harvesting system): on premises.

(7) ‘Shared’ equipment

Tap: on premises or off premises;

Well (or any other ground water): on premises or off premises;

Tank (no matter water tank or rainwater harvesting system): on premises or off premises.

(8) ‘Public’ / ‘communal’ / ‘village’ equipment

Network system (original source of piped water): on premises or off premises;

Tap / standpipe / fountain: off premises;

Well (or any other ground water): off premises;

Tank (no matter water tank or rainwater harvesting system): off premises.

(9) Purchased water / vended water / water vendor: improved or unimproved drinking-water, off premises.

(It could be purchased water from mobile seller or vending machine; tanker-truck; cart with small tank / drum; or could also be water refilling station.)

(10) Other ambiguous water sources without available definitions or any clear information were roughly considered as ‘undistinguishable’, which could be improved or unimproved drinking-water, could be on premises or off premises.

## Sanitation:

(1) Sanitation & Sewerage Disposal question sets

When multiple questions (sanitation & sewerage disposal) were applied, we assumed that 'sewage disposal' was also applied for toilet water if unspecified, and combined the sewage disposal categories with flush or pour-flush toilets. Therefore, generally ‘sewerage disposal’ does not provide any elimination / disposal related information for those toilets without flushing systems, such as pit latrines, bucket toilets, etc.

(2) Other ambiguous / non-standard categories of toilet without available definitions or any clear information were roughly considered as ‘undistinguishable’ sanitation.
